# Characteristics of persons refusing oral polio vaccine during the immunization plus days – Sokoto, Nigeria 2011

**DOI:** 10.11694/pamj.supp.2014.18.1.4187

**Published:** 2014-07-21

**Authors:** Abdulaziz Mohammed, Kabir Sabitu, Patrick Nguku, Emmanuel Abanida, Sadik Sheidu, Mahmood Dalhat, Raymond Dankoli, Saheed Gidado, Idris Suleiman

**Affiliations:** 1 Nigeria Field Epidemiology and Laboratory Training Program, Abuja, Nigeria; 2 Department of Community Medicine, Ahmadu Bello University, Zaria, Nigeria; 3 National Primary Health Care Development Agency, Abuja, Nigeria

**Keywords:** Immunization, OPV, Vaccine refusal, WPV, poliomyelitis, knowledge, attitude, noncompliance

## Abstract

**Introduction:**

Nigeria, the only African country endemic for wild poliovirus, adopted Immunization Plus Days (IPD) to eradicate polio. Refusal of oral polio vaccine (OPV) by heads of households is a significant challenge. In Sokoto state, we determined characteristics of heads of households refusing OPV during IPD in 2011.

**Methods:**

To evaluate reasons for refusals, we conducted a case control study among heads ofhouseholds accepting or refusing OPV vaccine. Noncompliant households were defined as households refusing OPV vaccination in last three rounds of IPDs while compliant households were those accepting vaccination. Interviewers administered a questionnaire to the heads of households to obtain information on socio-demographics, media habits, and knowledge of IPD.

**Results:**

Of the 121 (60 cases and 61 controls) interviews, 88 (73%) were from Sokoto north. Noncompliant heads of households were more likely to lack tertiary education (OR = 3.7, 95% CI, 1.6 - 9.2), believe that OPV is not safe (OR = 22, 95% CI, 7.1 - 76), lack access to functional radio (OR = 4.4, 95% CI, 1.4 - 15) and television (OR = 9.4, 95% CI, (1.9 - 63) andget information about IPD from town announcers (OR = 3.9, 95% CI, 1.3 - 12).

**Conclusion:**

We conclude that noncompliant heads of households compared to compliant heads of households had low level of education, lacked knowledge of immunization, and had negative attitude towards OPV. They get information about OPV from town announcers and lacked access to functional radio and television. We recommended training of town announcers in polio communication and use of key communication messages preceding every round of IPD.

## Introduction

In May 1988, the world health assembly resolved to eradicate poliomyelitis by the year 2000 [1]. Though this target was missed,the number of countries with endemic wild poliovirus (WPV) dropped to three, namely Nigeria, Pakistan, and Afghanistan in 2012. Furthermore,the annual incidence of polio reduced by 99% worldwide [2]. When the global effort to eradicate polio was launched in 1988, 350,000 children in 125 countries were permanently paralyzed by poliovirus each year, despite the availability of a cheap and effective oral polio vaccine (OPV) [3]. The Africa region adopted a three pronged immunization strategy targeting children < 5 years of age with OPV towards achieving a herd immunity of at least 80% necessary for interruption of WPV transmission [4]. These strategies included Supplemental Immunization Activities, achieving high level of routine immunization coverage, and house-to-housemop up campaigns carried out in areas where the virus is suspected to be circulating. Nigeria, the only African country still endemic for WPV,has increasingly used supplemental immunization activities to break the transmission of WPV because of the low routine immunization coverage [5]. The immunization plus days (IPD) is a supplemental immunization strategy used for mass immunization with OPV throughhouse-to-house vaccination aimed at eligible children irrespective of their previous vaccination status [4].

In 2003, some parents’ refusal of polio vaccination for their children led to the suspension of IPD in five states in northern Nigeria [6] andresurgence of WPV transmissionwith spread to 19 other polio free countries [7]. Though IPD srecommenced in 2004, refusal of vaccination by parents or guardians of children eligible for polio vaccination (which is also referred to as noncompliance) continued. Refusal of vaccination for eligible children by parents or caregivers has been demonstrated elsewhere. In a 2005 United States study offactors associated with refusal of childhood vaccination, parents who refused believed the efficacy of vaccines were low and had concerns about vaccine safety [8]. Studies have indicated that misperceptions of mass campaigns among caregivers could lead to non-vaccination of their children [8–15]. Between the periods January 2004 through 2008, Sokoto state of Nigeria reported 186 cases of polio [16]. In the January 2009 IPD, about 50% of monitored wards (the smallest political division) in Sokoto state had >10% missed children [17]. The independent monitoring board of the World Health Organization (WHO) concluded that 25% of all missed children in Nigeria were due to vaccine refusal (noncompliance) and that even if other problems of the eradication efforts in Nigeria are sorted out, the refusal issue alone is sufficient to undermine success [18]. We set out to determine the characteristics of heads of households refusing OPV (noncompliant) during IPDs in Sokoto state. Specifically, we wanted to determine the socio-demographic correlates, the knowledge, attitude, and sources of information of heads of households refusing OPV during IPD as compared with compliant households. We also assessed decision factors influencing heads of householdspreviously refusing OPV, to recommend how to minimize OPV refusal during IPD.

## Methods

**Study setting:** The study was conducted in Sokoto north and Sokotosouth local government areas of Sokoto state located in the Northwestern region of Nigeria in November 2011. Sokoto state shares an international boundary with Niger Republic to the north and two other Nigerian states (Kebbi and Zamfara states) to the south.

**Study design:** We conducted an unmatched case-control study in 2 local government areas in Sokoto state (Sokoto North and Sokoto South). Participants refusing vaccination werethe heads of householdswhere the parent or guardian of an eligible child refused OPV in the last three consecutive rounds of IPD. Participants for controls werethe nearest household to the right of the noncompliant household that allowed all its eligible children to be vaccinated in the past three rounds. Inclusion criteria were presence of heads of householdson the list of noncompliance for the last three rounds of IPD in 2011 andpresence of an eligible child (0-5 years) within the household. Exclusion criteriawere refusal of consent, absence of head of household after three visits, and relocation of the household outside the study area.

### Sample size determination

We calculated sample size calculations based on an estimated prevalence of poor knowledge of benefits of vaccination to be 50% in case households and 16% in control households. We used the formula below to calculate our sample size:

$$\text{n}(\text{each group})=\left({\text{p}}_{\text{0}}{\text{q}}_{\text{0}}+{\text{p}}_{\text{1}}{\text{q}}_{\text{1}}\right){\left({\text{z}}_{\text{1}}-\alpha /2+{\text{z}}_{\text{1}}-\alpha \right)}^{2}/{\left({\text{p}}_{\text{1}}-{\text{p}}_{\text{0}}\right)}^{2}$$

Where,

n = desired sample size in each group (cases and control)

p_0_ = Proportion of satisfactory knowledge of immunization in controls taken to be 16% [19, 20]

q_0_= 1- p_0_

p_1_ = Proportion of satisfactory knowledge of immunization among cases assumed to be 50% for maximum variability

q_1_=1- p_1_

z_1_-α/2 = Standard normal deviate (1.96) corresponding to 95% confidence interval

z_1_-α = Standard normal deviate (0.84) corresponding to a power of 80%

Therefore for this study 65 cases and 65 controls was interviewed.

**Sampling technique:** The sampling frame for the study included names of heads of households that appeared on the list of noncompliance for the last three rounds of IPD inSokoto north and Sokoto south local government. The local immunization officer usually compiles the list of noncompliant heads of households during implementation of the IPD. The list was further adjusted to reflect the inclusion and exclusion criteria for the study. The number of households that made the final sampling frame was 295. Systematic sampling technique was used to select the respondents to be interviewed for the study. This was done by dividing the sampling frame (295) with the calculated sample size for cases (65) to determine the sampling interval. The first household was selected from the sampling frame using a table of random numbers and using the sampling interval we selected subsequent respondents until the required sample size was reached.

**Study instrument:** We used a pre-designed,interviewer-administered questionnaire with sections on socio-demographics, knowledge and attitudes of heads of household on IPD and reasons for refusal of vaccination. We also had a section on respondents’ access to information pertaining to IPD. Interviewers were recruited, trained and supervised by the principal investigator.

**Data entry and analysis:** All data were entered into epi-Info. Data were checked for its completeness and clarity by field supervisors and on the spot correction were made. Quality control by re-interviewing one percent of the respondents was done. We used frequencies, proportions and tables to summarize our data and bivariate analysis was performed to identify factors associated with noncompliant heads of households. Odds ratio with 95% confidence intervals was used to determine statistically significant associations

**Ethical consideration:** We soughtpermission from the Nigeria federal ministry of health before onset of the study and the proposal was submitted to the Federal Neuro-Psychiatric Hospital ethical committee for approval. Participating households were required to give an informed consent before inclusion in the study. All potential subjects were presented with a consent form, which described the type of study being done, the purpose of the study, and the subject's rights as a participant in the study, including the right to confidentiality, and the right to withdraw from the study. Names were not included in any of the findings and no financial inducement was given to any respondent.

## Results

**Socio-demographic variables:** A total of 130 interviews were conducted out of which nine (6.9%) were disqualified due to incomplete answers. Five selected cases were replaced due to either relocation of the household out of the study area or absence of the head of household after three visits. All selected heads of households that were available consented to be interviewed. The socio-demographic characteristics of those who were disqualified were similar to those who were analyzed. Of the 121 valid questionnaires (60 noncompliant households and 61 compliant households), 88 (73%) were from Sokoto north local government while the rest were from Sokoto south. The mean age of noncompliant heads of households was 48 years while that of the compliant heads of households was 44 years. Most of the respondents were males (noncompliant 100% vs. compliant 89%), Muslim (noncompliant 94% vs. compliant 95%), and 62% of noncompliant heads of households had polygamous marriagecompared to 45% of the compliantheads of households (Table 1).

**Table 1:** Socio-demographic distribution of respondents by noncompliance/compliance in Sokoto, Nigeria – 2011

Socio-demographic	Noncompliant		Compliant	
	n	%	N	%
**Sex**				
Female	0	0.0	7	11.0
Male	60	100.0	54	89.0
**Religion**				
Islam Christianity	56	95.0	57	95.0
**Marital status**				
Single	1	2	0	0.0
Married	57	98	60	98
Widowed	0	0.0	1	2.0
**Level of education**				
Primary	4	7.0	5	8.0
Secondary	25	42.0	13	22.0
Tertiary	12	20.0	29	48.0
Quranic	19	31.0	13	22.0
**Occupation**				
Agriculture (Farming and fishing)	15	26.0	4	6.6
Agriculture (Nomadic pastoralist)	4	7.0	3	5.0
Legislator, senior official and professional	1	2.0	14	23.0
Student	3	5.0	4	6.6
Shop and market worker	19	33.0	25	41.0
Technicians and associate professionals	6	10.0	4	6.6
Unemployed	10	17.0	7	11.0
**Type of marriage**				
Monogamous	22	38	31	55
Polygamous	36	62	25	45

**Socio-demographic, knowledge and attitudecharacteristics of noncompliant households:** Noncompliant heads of households were more likely to lack tertiary education (OR = 3.7) compared to compliant heads of households (Table 2). Noncompliant heads of households were more likely to lack knowledge of the benefit of immunization (OR = 35) and to lack knowledge of the global effort to eradicate polio (OR = 19). Knowledge of BCG vaccine given at fixed post during IPD did not differ between the comparison groups. Noncompliant heads of households were more likely to believe that OPV is not safe (OR = 22), and that OPV can cause infertility (OR = 18) (Table 3).

**Table 2:** Socio-demographic associations of noncompliant/compliant heads of households in Sokoto, Nigeria – 2011

Variable	Noncompliant		Compliant		OR (95% CI)
	N	%	n	%	
**Age (years)**					0.8 (0.4 – 1.7)
= 40	22	37.0	26	43.0	
> 40	38	63.0	35	57.0	
**Religion**					1.0 (0.2 – 6.4)
Islam	56	95.0	57	95.0	
Christianity	3	5.0	3	5.0	
**Level of education**					3.7 (1.6 – 9.2)
No tertiary	48	80.0	31	52.0	
Tertiary	12	20.0	29	48.0	
**Type of marriage**					2.0 (0.9 – 4.6)
Polygamous	36	62.0	25	45	
Monogamous	22	38.0	21	55	

**Table 3:** Knowledge, attitude and media habit concerning immunization plus days of noncompliant/compliant heads of households in Sokoto, Nigeria – 2011

Factors	Noncompliant		Compliant		OR (95% CI)
Knowledge of IPD	n	%	n	%	
**Benefit of immunization**					35 (9.8 – 141)
No	46	92.0	14	24.6	
Yes	4	8.0	43	75.4	
**Vaccine preventable disease other than polio**					0.6 (0.2 – 1.8)
No	47	78.3	52	85.2	
Yes	13	21.7	9	14.8	
**Global nature of polio eradication**					19 (6.9 – 54)
No	46	76.7	9	14.8	
Yes	14	23.3	52	85.2	
**Age at which 1st dose of OPV is given**					16 (5.1 – 52)
No	35	58.3	5	8.2	
Yes	25	41.7	56	91.8	
**BCG given at health center during IPD**					2.1 (0.5 – 9)
No	56	93.3	53	86.9	
Yes	4	6.7	8	13.1	
**Attitude towards IPD**					
IS OPV Safe?					22 (7.1 – 76)
No	40	66.7	5	8.2	
Yes	20	33.3	56	91.8	
**Can OPV cause infertility**					18 (4.7 – 81)
No	29	48.3	3	4.9	
Yes	31	51.7	58	95.1	
**Source of information**					
Type of media					3.9(1.3 – 12)
Town Announcer	16	36.0	7	13.0	
Electronic media	28	64.0	48	87.0	
**Media access**					
**Access to functional radio**					4.4 (1.4 – 15)
No	17	28.0	5	8.0	
Yes	43	72.0	56	92.0	
**Access to functional TV**					9.4 (1.9 – 63)
No	14	24.0	2	3.0	
Yes	44	76.0	59	97.0	
**Access to newspaper**					1.4 (0.6 – 3.1)
No	40	67.0	36	59.0	
Yes	20	33.0	25	41.0	

**Figure 1:**
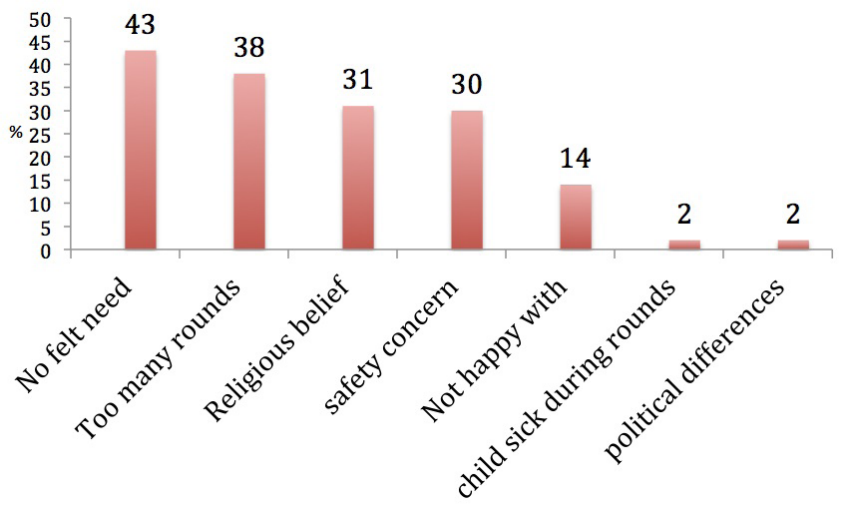
Reasons for OPV refusal among the noncompliant heads of households in Sokoto, Nigeria, 2011 (n = 60)

Access to information about IPDs and reasons for refusals among non-compliant heads of household: Noncompliant heads of household were more likely “To source” information concerning IPD from town announcers (OR = 3.9) and lack access to a functional radio (OR = 4.4) and television (OR = 9.4)(Table 3). The most frequent reason given for vaccine refusal among the noncompliant heads of households was “no felt need” (Figure 1).

**Decision factors influencing heads of household previously refusing OPV:** The heads of households were personally responsible for taking decision regarding OPV in 43 of 60 noncompliant heads of households and 37 of 61 compliant heads of household. Of the 61 compliant heads of household, 27 (44%) had never refused OPV in the past while 34 (56%) refused OPV at least once prior to last 3 rounds of IPD in 2011. Of the 34 who had previously refused OPV, 21 (62%) were convinced to start accepting OPV by traditional rulers and five (15%) by religious leaders.

## Discussion

In this study we found that the noncompliant heads of households compared to the compliant heads of households had lower education, lacked knowledge of benefit of immunization, and did not know the global nature of polio eradication. The noncompliant heads of householdsalso did not know the age of first dose of OPV and they also believed that OPV is not safe and that OPV can cause infertility. They also lacked assess to functional televisions and radios and mostly sourced their information about IPD from town announcers. Decisions about immunizationwere taken by the noncompliant head of households and the most common reasons for refusing OPV among the noncompliant head of households were “no felt need” and “too many rounds”. The traditional leaders were the most successful in positively influencing the decision to vaccinate. In our study, lacking a tertiary education was associated with noncompliant households. This may appear worrying for the polio eradication program in Nigeria, a country with adult literacy rate of about

57% and much lower (25 - 50%) for northern Nigeria [20]. However using polio communication messages, targeted at noncompliant heads of households, in terms of contents and methods of delivery may be beneficial. Low educational level has been associated with vaccine refusal inmany developing countries. In a Turkish study, mothers with higher educational background chose to fully immunize their children compared to those with lower education [12]. Low level of parents’ education was also found to be associated with non-vaccination during mass campaign in India [14]. In previous studies in Nigeria, the level of education was found to be important in accepting OPV during IPDin Zaria, northern Nigeria [6]. In developed countries, advanced educational status has been a factor in vaccine refusals [21].

Lacking the knowledge of benefit of immunization was a factor for OPV refusal during IPD. The knowledge of the benefit of immunization may be important especially when parents are required to immunize their children repeatedly as is the case with IPD. Parents might prioritize preventing other diseases such as measles compared to polio considered by them to be a rare disease. Caregivers’ apathy to immunization against a disease due to perceived low risk of infection had been demonstrated in the USA [22]. In a cross sectional study of determinants of immunization coverage in Zamfara state of Nigeria, the researchers found that parents’ knowledge of immunization was a significant determinant of immunization status of the child. Lack of knowledge of the global effort to eradicate polio was also a factor for OPV refusal in this study. The knowledge that polio eradication is not restricted to Nigeria alone but a global effort much like the smallpox mass campaign several years ago may give the process added credibility that might reduce OPV refusal. Understanding the gains of global eradication of polio could provide the rational for the focus on polio eradication by the government. There was a generally low knowledge of existence of vaccines in fixed post during IPD. The knowledge of availability of other vaccines in fixed post during IPD may help reduce the perception that polio is been singled out for mass campaign to the detriment of other childhood diseases. The fixed post has the added advantage of strengthening routine immunization. Ironically, the success of the polio eradication program in preventing paralysis among children < 5 years of age has decreased visibility of polio as a disease in many communities compared to measles and other vaccine preventable diseases.

We found that noncompliant heads of households believed that OPV was not safe and that it may cause infertility, a similar finding to that, in neighboring country of Cameroon [23]. The noncompliant heads of householdswere less likely to have access to functional television or radio. This might suggest that they were more likely to get their information from sources other than mass media. This has implication in the design of effective communication targeted at the noncompliant heads of households. The noncompliant heads of households were more likely to source their information about IPD from the town announcers. This might be due to the lower level of education among the noncompliant heads of households but may also point to the need to critically examine the communication skills of town announcersused during IPD campaigns. The polio eradication program in Nigeria may also consider training the town announcers on key communication messages. The finding that decision on immunization was mostly taken by the heads of household is significant for polio communication. Polio communication sessions targeted at heads of households done after work hours should be considered. The most common reasons given by noncompliant heads of households for refusing OPV were “no felt need” and “too many rounds”. The “too many round” reason may indicate genuine fatigue to the frequent rounds of IPD in Nigeria and may underlies the importance of ensuring that all impediments to achieving high quality IPDs is urgently addressed by the program. This may reduce low herd immunity to pockets of small areas allowing effective use of targeted mop up campaigns and less frequent large scale IPDs. An evaluation of polio mass campaign in India also found the most common reason given by parents for refusing vaccination to be “too many doses” [24]. No felt need had also been demonstrated as a reason for vaccine refusal [25].

The findings of this study are subject to the following limitations. Only two of the 23 local government areas in Sokoto state were studied therefore the findings can only be generalized to the two areas. Alsowe only studied urban local government areas and might have missed some of the peculiarities of rural populations. Despite these limitations we are confident that the findings of this study would be useful to Sokoto state as a whole and other parts of northern Nigeria that share similar socio-cultural practices to the two local governments used for the study.

## Conclusion

We conclude that characteristics of noncompliant heads of households in Sokoto state were low levels of education, lacking knowledge of immunization, and had negative attitude towards OPV. They get information about OPV from town announcers and lacked access to functional radio and television. No felt need and too many rounds were the main reason for refusing OPV. Traditional leaders were successful in positively influencing previously noncompliant heads of households to accept OPV. We recommendedcommunity dialogue preceding every round of IPD with refined key communication messages such as “polio eradication is a global effort” and the need for several rounds of OPV vaccination and benefits of immunization targeted at the noncompliant heads of households. This should involve the use ofalternative methodsof communications such as mobile text messages and village play to get to the noncompliant heads of households. We also recommended evaluation of the communication messages of the town announcers. Finally we recommend that mosquito nets, de-worming tablets and other child survival strategies that were supposed to be bundled with OPV during IPD be scaled up.
